# Validation of Diagnostic Codes to Identify Carbon Monoxide Poisoning in Taiwan’s Claims Data

**DOI:** 10.3389/fphar.2022.882632

**Published:** 2022-06-15

**Authors:** Min-Ying Chiang, Shih-Chieh Shao, Shu-Chen Liao

**Affiliations:** ^1^ Department of Emergency Medicine, Keelung Chang Gung Memorial Hospital, Keelung, Taiwan; ^2^ College of Medicine, Chang Gung University, Taoyuan, Taiwan; ^3^ Department of Pharmacy, Keelung Chang Gung Memorial Hospital, Keelung, Taiwan; ^4^ School of Pharmacy, Institute of Clinical Pharmacy and Pharmaceutical Sciences, College of Medicine, National Cheng Kung University, Tainan, Taiwan

**Keywords:** validation, carbon monoxide poisoning, misclassication, database research, international classification of diseases codes

## Abstract

**Purpose:** Previous studies identified the study cohort or outcome of carbon monoxide poisoning (COP) by using the relevant disease diagnosis codes in secondary databases, but the validity of diagnosis codes of COP is unclear in such secondary databases. This study aimed to evaluate the accuracy of case definitions for COP using the International Classification of Diseases, Ninth Revision, Clinical Modification (ICD-9-CM) and Tenth Revision, Clinical Modification (ICD-10-CM) diagnosis codes in Taiwan’s health insurance claims data.

**Methods:** We selected a 10% simple random sample from an original cohort of patients newly undergoing carboxyhemoglobin (COHb) testing under any clinical diagnosis at four Chang Gung Memorial Hospitals in Taiwan during 2011–2020. Two clinical doctors independently ascertained the COP diagnosis by reviewing the medical records as the reference standard. We estimated the sensitivity, specificity, positive predictive value (PPV), and negative predictive value (NPV) of various case definitions (e.g., ICD-9-CM code, ICD-10-CM code and the uses of hyperbaric oxygen therapy) in the claims data.

**Results:** We randomly selected 557 cases from the original cohort of 5,571 cases newly receiving COHb test in the study hospitals. We found 90, 35, and 9 cases were true-positive, false-positive, and false-negative episodes of COP, respectively, among 278 cases with an ICD-9-CM code of 986. A further 111, 34, and 6 cases were true-positive, false-positive, and false-negative episodes of COP, respectively, among 279 cases with an ICD-10-CM code of T58. Using ICD-9-CM codes, the sensitivity, specificity, PPV and NPV for COP were 90.9, 80.4, 72, and 94.1%, respectively. Using ICD-10-CM codes they were 94.9, 79, 76.6, and 95.5%, respectively. PPV typically increased when COP was the primary diagnosis and could reach 100% if patients with ICD-CM code 986 or T58 also received hyperbaric oxygen therapy during hospitalization.

**Conclusion:** The COP-related ICD-CM codes alone did not accurately identify COP patients, but accuracy improved after including oxygen therapy data with the ICD-CM codes in Taiwan’s claims data.

## 1 Introduction

The worldwide incidence of carbon monoxide poisoning (COP) is estimated at 137 cases per million over the past two decades (Mattiuzzi and Lippi, 2020). In the United States, COP is the second most common non-medicinal poisoning death, resulting in 1.48 deaths per million people annually (Sircar et al., 2015). In addition to COP-related mortality in the acute stage, COP can also result in severe morbidities during the recovery stage, such as delayed neuropsychiatric sequelae. The causes of COP include intentional (e.g., suicide attempt), unintentional (e.g., indoor activity with poor ventilation) and occupational factors (e.g., fuel burning furnaces, or gasoline-powered generators). Specifically, during the current COVID-19 pandemic, people have increased indoor activities or developed psychological disorders during long periods of home quarantine (Dominguez-Amarillo et al., 2020; Jin et al., 2021), which may increase the incidence of intentional and unintentional COP, requiring much clinical attention.

Many studies have been published attempting to better understand the epidemiological features, risk factors, clinical outcomes and treatment effectiveness of COP using secondary data sources (e.g., electronic medical records, healthcare data, health insurance claims data or administrative data) (National Health Insurance Administration and Welfare, 2014; Huang et al., 2017; Liao et al., 2019; Pan et al., 2019; Huang et al., 2021; Wei et al., 2021). For example, based on Taiwanese claims data, Huang CC et al. found that COP was associated with higher risks of congestive heart failure (Huang et al., 2021). Stearns D et al., using US healthcare data, indicated the rate of hospitalizations for unintentional non fire-related COP did not change during 2003–2013 in the United States (Stearns and Sircar, 2019). Like most studies analyzing secondary databases, study investigators identified COP by using the relevant disease diagnosis codes. However, the impact of misclassification bias may threaten the study validity if the accuracy of disease diagnosis codes is unclear in studies using such secondary databases.

To construct and validate case definition algorithms for COP, this study reviewed electronic medical records data to evaluate the accuracy of International Classification of Diseases, Ninth Revision, Clinical Modification (ICD-9-CM) and Tenth Revision, Clinical Modification (ICD-10-CM) diagnosis codes used in Taiwan’s health insurance claims data.

## 2 Methods

### 2.1 Study Settings

This study was based in the four Chang Gung Memorial Hospitals (i.e., Keelung, Taoyuan, Linkou and Taipei branches) in Taiwan, covering approximately 14% of inpatients in northern Taiwan (Shao et al., 2019a). The study protocol has been approved by the Institutional Review Board of the Chang Gung Medical Foundation (IRB NO: 202100519B0). The requirement for informed consent was waived due to the retrospective design.

### 2.2 Study Cohort

This study used electronic medical records data from Chang Gung Memorial Hospitals, and claims data reported to the National Health Insurance Administration, retrieved from the hospital information system. Several diagnosis codes, including those for myocardial infarction, ischemic stroke and heart failure and cerebral venous sinus thrombosis, have been validated for this data source (Shao et al., 2019b; Liao et al., 2022). Since carboxyhemoglobin (COHb) levels are measured in all suspected cases of COP, we first identified all patients who had received COHb tests during 2011–2020. If patients received multiple COHb tests during the study period, we only included the first result from each patient. In addition, COHb is often used to exclude multiple clinical severe disease, such as large infarction, intracranial hemorrhage, epilepsy, life-threatening cardiac arrhythmia, and acute coronary syndrome. The study cohort was then selected from this group using a 10% simple random sampling approach via SAS Enterprise Guide 7.13 (SAS Institute, Inc., Cary, NC, United States) without any specific stratification to select a probability sample from the full cohort data. We subsequently critically reviewed their electronic medical records and retrieved their discharge diagnosis based on ICD-9-CM or ICD-10-CM codes. The steps for validation in this present study were similar to those of other, previous validation studies (Ceder et al., 2021; Ye et al., 2022).

### 2.3 Ascertainment of Carbon Monoxide Poisoning

The accurate diagnosis of COP is complicated since it may require data about patients’ exposure history and clinical signs or symptoms, in addition to the COHb levels. According to the guidance on COP from the Centers for Disease Control and Prevention (Centers for Disease Control and Prevention, 2020), we pre-specified the study protocol using a data collection form for related information to diagnose COP. Two experienced medical doctors (SCL, clinical toxicologist and MYC, emergency physician) independently performed data collection and reviewed the electronic medical records from the included patients. Any discrepancy was resolved by full discussion. We describe the detailed steps for COP case confirmation as follows: First, we obtained the COHb levels, and evaluated their history of exposure to CO (e.g., charcoal burning, fire, cluster exposure, etc.). Second, we assessed their initial symptoms (e.g., dizziness, nausea, vomiting, fatigue, shortness of breath, ataxia, altered mental status, coma, etc.) and signs (e.g., hypotension, arrhythmias, myocardial ischemia, metabolic acidosis, etc.) to determine if they were consistent with COP (Hampson et al., 2012; Liao et al., 2018; Centers for Disease Control and Prevention, 2020). Finally, we retrieved image reports such as computed tomography or magnetic resonance imaging, looking for any possible hypoxic-ischemic encephalopathy caused by COP (O'Donnell et al., 2000; Hegde et al., 2011).

**FIGURE 1:**
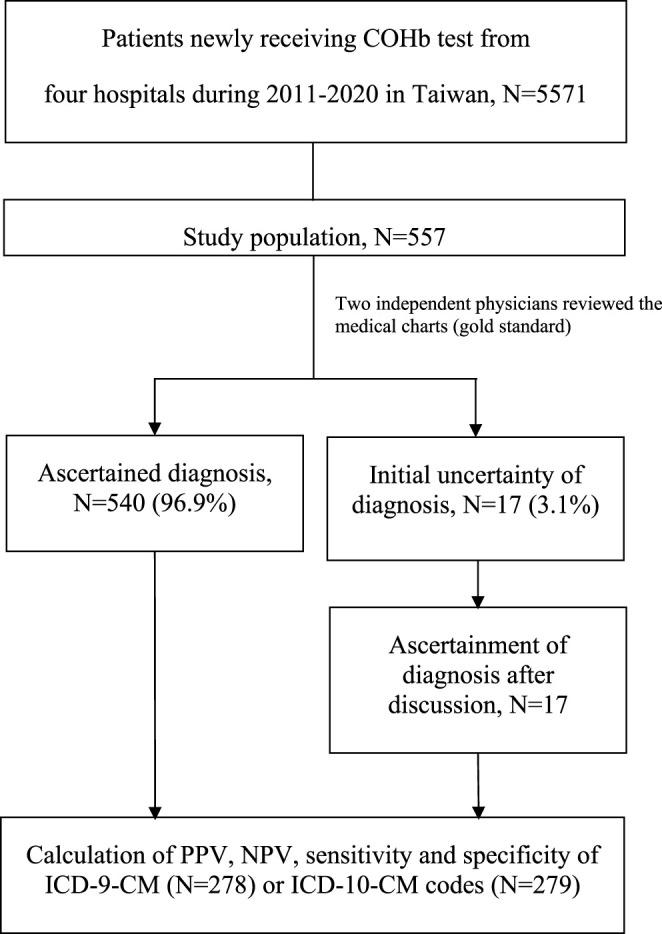
Process of case ascertainment.

After review of the electronic medical records, the COP cases were confirmed if 1) patients had COHb levels over 2% for non-smokers and 9% for smokers with or without CO exposure history or 2) patients had typical symptoms and signs, especially hypoxic-ischemic encephalopathy as shown by imaging, with or without CO exposure history (Hampson et al., 2012; Centers for Disease Control and Prevention, 2020). For some patients transferring to our hospitals with normal COHb levels, we based our COP judgment on the aforementioned information. Specifically, the use of oxygen or hyperbaric oxygen therapy was not included among clinical criteria to define true COP cases. In cases of disagreement between the reviewers, consensus on the final ascertainment was reached by discussion. In the case of false positives, the main diagnosis was presented. Figure 1 shows the process of case ascertainment.

### 2.4 Data Analyses

We employed several different case definitions to identify COP cases, including: 1) an ICD-9-CM code of 986 (Toxic effect of carbon monoxide) or an ICD-10-CM code of T58 (Toxic effect of carbon monoxide) as the primary diagnosis; 2) code 986 or T58 as the primary or secondary diagnosis; 3) code 986 or T58 as the primary, secondary, or tertiary diagnosis; and 4) code 986 or T58 in any field of diagnosis. The sensitivity, specificity, positive predictive value (PPV), and negative predictive value (NPV) were calculated and their 95% confidence intervals (CIs) for binomial proportions were estimated. To increase the accuracy of case definitions, we also combined the code records with records of specific treatment for COP (e.g., the use of oxygen therapy or hyperbaric oxygen therapy). Finally, we calculated the COP incidence (cases per 10,000 patient visits in the emergency room) before and after PPV adjustment. Data analyses were performed using SAS Enterprise Guide 7.13 (SAS Institute, Inc., Cary, NC, United States).

## 3 Results

We identified a total of 5,571 patients who received at least one COHb test at one of the four study hospitals in the period from 2011 to 2020. After 10% simple random sampling, the electronic medical records of 557 patients remained for review. Primary demographics of the full cohort (*n* = 5571) and the 10% simple random sampling sub-cohort (*n* = 557) were presented in Supplementary Table S1. Initial agreement on the COP diagnosis was high between the two reviewers (540/557: 96.9%), and the remaining 17 cases with conflicting judgements were resolved after full discussion between the reviewers (Figure 1). Of the 216 COP patients, most (*n* = 157, 72.7%) were discharged from the emergency department. Of the 341 non-COP patients, most (*n* = 194, 56.9%) were admitted to hospitals for further care. Demographic and clinical data of included patients with and without validated COP are presented in Supplementary Table S2.

Of the 557 reviewed patients, 278 were coded using ICD-9-CM diagnostic codes while 279 were coded using ICD-10-CM diagnostic code. Table 1 shows the validity of various case definitions to identify cases of COP. Among the 278 cases with an ICD-9-CM code of 986, 90, 35, and 9 were determined to be true-positive, false-positive, and false-negative episodes of COP, respectively, resulting in a PPV of 72% and sensitivity of 90.9%. The NPV and specificity for COP were 94.1 and 80.4%, respectively. Among the 279 cases with an ICD-10-CM code of T58, 111, 34, and 6 were determined to be true-positive, false-positive, and false-negative episodes of COP, respectively, resulting in a PPV of 76.6% and a sensitivity of 94.9%. The reasons for the false-positive determinations of COP are listed in Table 2. Among the false-positive cases (*n* = 69), we found the most frequent reason for miscoding was burns (*n* = 21), followed by other substance poisoning (*n* = 9) and peripheral vertigo (*n* = 6).

**TABLE 1:** Accuracy of different case definitions to identify carbon monoxide poisoning cases.

	TP, n	FP, n	FN, n	PPV, %	NPV, %	Sensitivity, %	Specificity, %
				(95% CI)	(95% CI)	(95% CI)	(95% CI)
Case definitions (ICD-9-CM codes)							
986 as primary diagnosis	74	0	25	100 (95.1–100)	87.7 (82.4–91.9)	74.7 (65–82.9)	100 (98–100)
986 as primary or secondary diagnosis	83	17	16	83 (74.2–89.8)	91 (85.8–94.8)	83.8 (75.1–90.5)	90.5 (85.2–94.4)
986 as primary, secondary, or tertiary diagnosis	87	22	12	79.8 (71.1–86.9)	92.9 (87.9–96.3)	87.9 (79.8–93.6)	87.7 (82–92.1)
986 in any field of diagnosis	90	35	9	72 (63.3–79.7)	94.1 (89.1–97.3)	90.9 (83.4–95.8)	80.4 (73.9–86)
Case definitions (ICD-10-CM codes)							
T58 as primary diagnosis	95	2	22	97.9 (92.8–99.8)	87.9 (82.3–92.3)	81.2 (72.9–87.8)	98.8 (95.6–99.9)
T58 as primary or secondary diagnosis	105	26	12	80.2 (72.3–86.6)	91.9 (86.3–95.7)	89.7 (82.8–94.6)	84 (77.4–89.2)
T58 as primary, secondary, or tertiary diagnosis	107	30	10	78.1 (70.2–84.7)	93 (87.4–96.6)	91.5 (84.8–95.8)	81.5 (74.6–87.1)
T58 in any field of diagnosis	111	34	6	76.6 (68.8–83.2)	95.5 (90.5–98.3)	94.9 (89.2–98.1)	79 (71.9–85)

TP, true positive; FP, false positive; FN, false negative; ICD-9-CM code, International Classification of Diseases Code, 9^th^ Revision, Clinical Modification; ICD-10-CM, International Classification of Diseases, 10th Revision, Clinical Modification; PPV, positive predictive value; NPV, negative predictive value; CI, confidence interval.

**TABLE 2:** Reasons for false-positive carbon monoxide poisoning cases.

False positive	ICD-9-CM (*n* = 35)	ICD-10-CM (*n* = 34)
Burn	8	13
Other substance poisoning	5	4
Peripheral vertigo	3	3
Pneumonia	2	1
Dizziness	2	2
Cerebrovascular accident	2	0
Intracerebral hemorrhage	2	0
Psychogenic	2	1
Syncope	2	3
Metabolic encephalopathy	2	0
Seizure	2	1
Brain tumor	1	0
Migraine	1	0
Trauma	1	0
Chest pain	0	1
Acute coronary syndrome	0	1
Out-of-hospital cardiac arrest	0	1
Hypoglycemia	0	1
Septic shock	0	1
Cyanosis	0	1

ICD-9-CM code,International Classification of Diseases Code, 9th Revision, Clinical Modification; ICD-10-CM, International Classification of Diseases, 10th Revision, Clinical Modification.

When the ICD-9-CM code of 986 and ICD-10-CM code of T58 served as the primary diagnosis to identify COP, the PPVs were 100 and 97.9%, respectively. However, using this definition would drop 17.8 and 14.4% of COP cases, respectively, from the initial cohort, given the sensitivity of 74.7 and 81.2%, respectively, as codes for other critical medical conditions may have been used for the primary diagnosis. We found a total of 25 and 22 COP cases without ICD-9-CM code and ICD-10-CM code in the primary position, respectively, probably because these cases were comorbid with more severe diseases, either from complications of COP (e.g., general weakness: *n* = 1, dizziness: *n* = 1, consciousness change: *n* = 1 for those with ICD-9-CM code; and cardiac arrest or respiratory failure: *n* = 2, syncope: *n* = 1, sepsis: *n* = 1 with ICD-10-CM code) or other clinically critical conditions (e.g., burns of face or respiratory tract: *n* = 13, mixed intoxication: *n* = 4, sepsis: *n* = 2 with ICD-9-CM code; and burns of face or respiratory tract: *n* = 11, pulmonary embolism: *n* = 1, sepsis: *n* = 1 for those with ICD-10-CM code). Expanding the case definitions of COP to the secondary, tertiary, or any field of diagnosis, decreased the PPV of each definition, while the number of identified COP cases increased. By contrast, the clinical performance of specificity, which represents the ability to exclude COP, was much more precise as the identifying strategy. Of the 169 true COP cases coded by the primary diagnosis position, most (*n* = 137, 81.7%) were discharged from the emergency department. Of the 47 true COP cases coded by other diagnosis positions, most (*n* = 27, 57.4%) were admitted to hospitals for further care. Disposition analyses of the true COP cases by primary diagnosis and other diagnosis positions are presented in Supplementary Table S3.

Tables 3, 4 show the performance of the ICD-CM codes with the inclusion of oxygen therapy or hyperbaric oxygen therapy to identify COP. While the PPV and specificity of this definition could reach 100%, the COP case numbers were less than half of those using ICD-CM codes only.

**TABLE 3:** Accuracy of combined case definitions plus oxygen therapy to identify carbon monoxide poisoning cases.

	TP, n	FP, n	FN, n	PPV, %	NPV, %	Sensitivity, %	Specificity, %
				(95% CI)	(95% CI)	(95% CI)	(95% CI)
Case definitions (ICD-9-CM codes + Oxygen therapy)
986 as primary diagnosis	71	0	28	100 (94.9–100)	86.5 (81.1–90.8)	71.7 (61.8–80.3)	100 (98–100)
986 as primary or secondary diagnosis	79	8	20	90.8 (82.7–96)	89.5 (84.3–93.5)	79.8 (70.5–87.2)	95.5 (91.4–98.1)
986 as primary, secondary, or tertiary diagnosis	82	10	17	89.1 (80.9–94.7)	90.9 (85.8–94.6)	82.8 (73.9–89.7)	94.4 (90–97.3)
986 in any field of diagnosis	85	20	14	81 (72.1–88)	91.9 (86.8–95.5)	85.9 (77.4–92.1)	88.8 (83.3–93)
Case definitions (ICD-10-CM codes + Oxygen therapy)
T58 as primary diagnosis	91	2	26	97.8 (92.5–99.7)	86 (80.2–90.7)	77.8 (69.2–84.9)	98.8 (95.6–99.9)
T58 as primary or secondary diagnosis	99	13	18	88.4 (81–93.7)	89.2 (83.5–93.5)	84.6 (76.8–90.6)	92 (86.7–95.7)
T58 as primary, secondary, or tertiary diagnosis	101	16	16	86.3 (78.7–92)	90.1 (84.5–94.3)	86.3 (78.7–92)	90.1 (84.5–94.3)
T58 in any field of diagnosis	105	19	12	84.7 (77.1–90.5)	92.3 (86.9–95.9)	89.7 (82.8–94.6)	88.3 (82.3–92.8)

TP, true positive; FP, false positive; FN, false negative; ICD-9-CM code, International Classification of Diseases Code, 9th Revision, Clinical Modification; ICD-10-CM, International Classification of Diseases, 10th Revision, Clinical Modification; PPV, positive predictive value; NPV, negative predictive value; CI, confidence interval.

**TABLE 4:** Positive predictive value of combined case definitions plus hyperbaric oxygen therapy to identify carbon monoxide poisoning cases.

	TP, n	FP, n	FN, n	PPV, %	NPV, %	Sensitivity, %	Specificity, %
				(95% CI)	(95% CI)	(95% CI)	(95% CI)
Case definitions (ICD-9-CM codes + Hyperbaric oxygen therapy)							
986 as primary diagnosis	41	0	58	100 (91.4–100.0)	75.5 (69.5–80.9)	41.4 (31.6–51.8)	100 (98–100.0)
986 as primary or secondary diagnosis	42	0	57	100 (91.6–100.0)	75.8 (69.9–81.2)	42.4 (32.6–52.8)	100 (98–100.0)
986 as primary, secondary, or tertiary diagnosis	43	0	56	100 (91.8–100.0)	76.2 (70.2–81.5)	43.4 (33.5–53.8)	100 (98–100.0)
986 in any field of diagnosis	44	0	55	100 (92–100.0)	76.5 (70.5–81.8)	44.4 (34.5–54.8)	100 (98–100.0)
Case definitions (ICD-10-CM codes + Hyperbaric oxygen therapy)							
T58 as primary diagnosis	50	0	67	100 (92.9–100.0)	70.7 (64.4–76.6)	42.7 (33.6–52.2)	100 (97.8–100.0)
T58 as primary or secondary diagnosis	51	0	66	100 (93.0–100.0)	71.1 (64.7–76.9)	43.6 (34.5–53.1)	100 (97.8–100.0)
T58 as primary, secondary, or tertiary diagnosis	52	0	65	100 (93.2–100.0)	71.4 (65–77.2)	44.4 (35.3–53.9)	100 (97.8–100.0)
T58 in any field of diagnosis	52	0	65	100 (93.2–100.0)	71.4 (65–77.2)	44.4 (35.3–53.9)	100 (97.8–100.0)

TP, true positive; FP, false positive; FN, false negative; ICD-9-CM code, International Classification of Diseases Code, 9^th^ Revision, Clinical Modification; ICD-10-CM, International Classification of Diseases, 10th Revision, Clinical Modification; PPV, positive predictive value; NPV, negative predictive value; CI, confidence interval.

**FIGURE 2:**
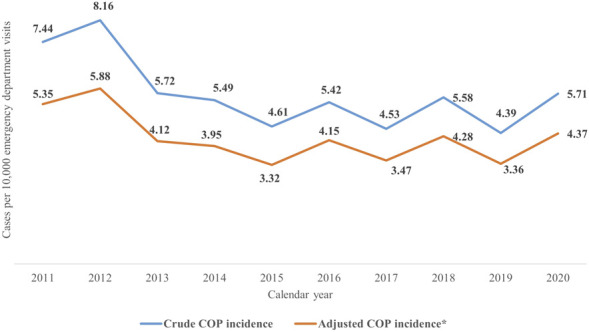
The crude and adjusted incidence of COP during 2011–2020. COP, carbon monoxide poisoning. *The crude incidence of COP multiplied by the PPV (2011–2015: 72%; 2016–2020: 76.6%) equals to the adjusted incidence of COP.

Figure 2 shows the crude and adjusted incidence of COP. The adjusted incidence of COP ranged from 5.35 to 3.32, which were calculated by using the PPV of 72% from 2011 to 2015; the adjusted incidence of COP ranged from 4.37 to 3.36, which were calculated by using the PPV of 76% from 2016 to 2020.

## 4 Discussion

Using a multi-institutional electronic medical records dataset, we found that COP-related ICD-CM codes alone could not accurately identify patients with COP in Taiwan’s claims data when they were used in any field of diagnosis. Of note, the results from ICD-9-CM codes and ICD-10-CM codes showed similar validities. However, the PPVs could be increased to near 100% by restricting ICD-CM codes to the primary diagnosis or including oxygen therapy, but this strategy may lead to the loss of possible COP cases. For patients with complications of COP or other clinically critical conditions comorbid with COP, such as full-thickness burns and respiratory failure, the COP-related ICD-CM codes were coded in the non-primary positions.

To the best of our knowledge, no published literature has hitherto indicated the validity of COP diagnosis codes in secondary data sources. However, a report from Ball LB et al. suggests there were some false positive cases of COP when they used ICD-9-CM codes of 986 as the case definition, potentially leading to overestimation of the epidemiological features of COP (Ball et al., 2005). Consistent with previous reports, we found the false positive rate of COP when using ICD-9-CM codes of 986 or ICD-10-CM codes of T58 in any field of diagnosis was about 20%. Compared to the COP group, the non-COP group had more severe conditions leading to higher admission rate. Based on our reviews of the electronic medical charts, we found the explanation may be that non-COP patients with other acute diseases (e.g., burns, other substance poisoning and initially unknown etiology of consciousness changes) presented with unspecific signs or symptoms similar to those of COP, and therefore clinical doctors would routinely check their COHb levels to clarify the COP status.

The adjusted incidence of COP in our study ranges from 3.32 to 5.35 per 10,000 patient visits in the emergency department, which is higher than that reported in worldwide statistics (1.37 cases per 10,000). A possible explanation may lie in cultural differences between Taiwan and other countries. In Taiwan, consumers have become accustomed to using gas powered water heaters, compared to electric water heaters in many Western countries (Wang et al., 2021). In addition, some intentional COP cases in Taiwan result from attempted suicide by indoor charcoal burning, which is rarely used as a suicide method in Western countries (Ajdacic-Gross et al., 2008; Lin et al., 2010).

The diagnosis of COP represented by both ICD-9-CM and ICD-10-CM codes is “Toxic effect of carbon monoxide”, so the validity of these two coding systems is important to know, in order to evaluate the effect of the transition in the diagnostic coding system on the identification of COP. Previous study from Quan H et al. indicates similar validity of coding for major diseases for both ICD-9-CM and ICD-10-CM codes, based on Canadian administrative data (Quan et al., 2008). Consistent with the previous report, we found the PPV, NPV, sensitivity and specificity of COP diagnosis were all similar for both the ICD-9-CM and ICD-10-CM codes. The implementation of ICD-10-CM coding did not lead to changes in the coding of COP, and potential bias from the coding system transition may be minor.

Based on our study results, we have two suggestions for future clinical researchers who are interested in COP studies using Taiwan’s claim data. First, if the researchers aim to capture the greatest number of patients with a true diagnosis of COP, for example, to evaluate the outcomes of COP, we recommend case definitions with moderate-to-high PPVs, such as using ICD-9-CM code 986 or ICD-10-CM code T58 in any field of diagnosis. Second, if researchers aim to analyze the outcomes of other primary causes (e.g., burns) for COP, we recommend case definitions using ICD-9-CM code 986 or ICD-10-CM code T58 as a non-primary diagnosis.

The major advantage of using electronic medical records reviews to validate the diagnosis codes of COP retrieved from the claims data in this study is that we could obtain broad clinical data to provide important demographic information about the validated cases. However, our study has several limitations. First, COHb tests or hyperbaric oxygen therapies are not available in some institutions, and patients with COP might be transferred to other larger-scale hospitals for further diagnoses or care. Our study hospitals are responsible for critical care in northern Taiwan, so our study sample also included those who were transferred from other hospitals. Second, smoking history information may be unclear in some COP cases, especially in those not fully conscious. Third, COHb levels as the criterion for the diagnosis of COP may vary between different guidelines (Raub et al., 1999; Weaver, 2009; Hampson and Dunn, 2012; Hampson et al., 2012; Centers for Disease Control and Prevention, 2020). However, we confirmed the COP diagnoses not only by COHb levels but also by CO exposure clues, clinical signs and symptoms. Finally, the validity of our COP case definitions applies to our four study hospitals, and the results may not generalize to all hospitals in Taiwan. However, we consider our findings to be representative since the study data was extracted from different hospital levels (e.g., district hospitals, regional hospitals and medical centers) and covered about 14% of inpatients in northern Taiwan. Further multi-center validation studies to replicate our findings are suggested.

## 5 Conclusion

The COP-related ICD-CM codes in Taiwan’s claims data may not have accurately identified patients with COP, but the accuracy could be improved by including data on oxygen therapy with the ICD-CM codes. Our findings with regard to PPV, NPV, sensitivity and specificity of different case definitions using ICD-CM codes and oxygen therapy data may provide a fundamental reference for future claims-based research related to COP in Taiwan.

## Data Availability

Data sharing is not applicable as study data analysis were performed on a statistics server through remote access in Chang Gung Medical Foundation in Taiwan to ensure the data privacy and safety. Further enquiries can be made to SCL, ermdsusan@gmail.com.
