# Supplementary material for: Validation of Diagnostic Codes to Identify Carbon Monoxide Poisoning in Taiwan’s Claims Data
Source: Front Pharmacol. 2022 Jun 15;13:882632. doi: 10.3389/fphar.2022.882632 (PMC9240270; doi:10.3389/fphar.2022.882632)
Supplement: Supplementary file 1 [file Table1.DOCX]

**Supplementary Table 1. Primary demographics of the full cohort and the simple random sampling sub-group**

|  | Full cohort (N=5571) | Sub-group (N=557) |
| --- | --- | --- |
| Mean age, years | 35.9 ± 22.6 | 36.4 ± 23.3 |
| Female, % | 42.7% | 46.0% |
| Presenting year |  |  |
| 2011 | 10.9% | 11.0% |
| 2012 | 11.4% | 10.4% |
| 2013 | 8.9% | 10.1% |
| 2014 | 9.3% | 10.1% |
| 2015 | 8.9% | 8.4% |
| 2016 | 11.1% | 12.6% |
| 2017 | 9.9% | 8.1% |
| 2018 | 11.2% | 12.4% |
| 2019 | 9.4% | 8.6% |
| 2020 | 8.9% | 8.4% |

Continuous data were expressed as mean ± standard deviation, whereas categorical data were presented as the number (proportion).

| **Supplementary Table 2. Demographic and clinical data of patients with and without validated COP** | | |
| --- | --- | --- |
|  | COP (N=216) | Non-COP (N=341) |
| Gender |  |  |
| Male | 102 (47.2%) | 199 (58.4%) |
| Female | 114 (52.8%) | 142 (41.6%) |
| Age, years | 36.3 ± 18.0 | 36.4 ± 26.0 |
| Psychiatric comorbidity history |  |  |
| Yes | 32 (14.8%) | 15 (4.4%) |
| No | 184 (85.2%) | 326 (95.6%) |
| CO exposure history |  |  |
| Yes | 157 (72.7%) | 89 (26%) |
| Charcoal burning | 71 (45.2%) | 2 (2.2%) |
| Water heater incomplete combustion or incorrect use of furnace | 25 (15.9%) | 4 (4.4%) |
| Fires | 44 (28.0%) | 72 (80.9%) |
| Others | 17 (10.8%) | 11 (12.4%) |
| No | 59 (27.3%) | 252 (74%) |
| Smoking history |  |  |
| Yes | 43 (19.9%) | 67 (19.6%) |
| No or quit | 89 (41.2%) | 224 (65.7%) |
| Unknown | 84 (38.9%) | 50 (14.7%) |
| Glasgow Coma Scale | 13.8 ± 3.0 | 13.7 ± 3.0 |
| Attempted suicide | 69 (31.9%) | 12 (3.5%) |
| Transient loss of consciousness | 97 (44.9%) | 73 (21.4%) |
| COHb, % (measured at first medical institution) | 15.3 ± 14.5 | 1.6 ± 1.2 |
| Modified Poisoning Severity Score | 2.7 ± 1.0 | - |
| Disposition |  |  |
| Discharge from ED | 157 (72.7%) | 106 (31.1%) |
| General ward admission | 24 (11.1%) | 40 (11.7%) |
| ICU admission | 33 (15.3%) | 154 (45.2%) |
| Death | 2 (0.9%) | 41 (12.0%) |

CO: carbon monoxide; COP: carbon monoxide poisoning; COHb: carboxyhemoglobin; ED: emergency department; ICU: intensive care unit.

Continuous data were expressed as mean ± standard deviation, whereas categorical data were presented as the number (proportion).

**Supplementary Table 3. Dispositions of the true COP cases coded by primary and other diagnosis positions.**

|  | True COP cases coded by primary diagnosis position (N=169) | True COP cases coded by other diagnosis positions (N=47) |
| --- | --- | --- |
| Death | 1 (0.6%) | 1 (2.1%) |
| Admission (ICU and general ward) | 30 (17.8%) | 27 (57.4%) |
| ICU | 12 (7.1%) | 21 (44.7%) |
| General ward | 18 (10.7%) | 6 (12.8%) |
| Discharge from ED | 138 (81.7%) | 19 (40.4%) |

COP: carbon monoxide poisoning; ICU: intensive care unit; ED: emergency department.

Continuous data were expressed as mean ± standard deviation, whereas categorical data were presented as the number (proportion).
